# Direct observation methods: A practical guide for health researchers

**DOI:** 10.1016/j.pecinn.2022.100036

**Published:** 2022-04-04

**Authors:** Gemmae M. Fix, Bo Kim, Mollie A. Ruben, Megan B. McCullough

**Affiliations:** a VA Center for Healthcare Organization and Implementation Research, Bedford and Boston, MA, USA; b General Internal Medicine, Boston University School of Medicine, Boston, MA, USA; c Department of Psychiatry, Harvard Medical School, Boston, MA, USA; d Department of Psychology, University of Maine, Orono, ME, USA; e Department of Public Health, University of Massachusetts Lowell, Lowell, MA, USA

**Keywords:** Direct Observation, Methods, Qualitative Methods, Ethnography, Health Services Research

## Abstract

**Objective:**

To provide health research teams with a practical, methodologically rigorous guide on how to conduct direct observation.

**Methods:**

Synthesis of authors’ observation-based teaching and research experiences in social sciences and health services research.

**Results:**

This article serves as a guide for making key decisions in studies involving direct observation. Study development begins with determining if observation methods are warranted or feasible. Deciding what and how to observe entails reviewing literature and defining what abstract, theoretically informed concepts look like in practice. Data collection tools help systematically record phenomena of interest. Interdisciplinary teams--that include relevant community members-- increase relevance, rigor and reliability, distribute work, and facilitate scheduling. Piloting systematizes data collection across the team and proactively addresses issues.

**Conclusion:**

Observation can elucidate phenomena germane to healthcare research questions by adding unique insights. Careful selection and sampling are critical to rigor. Phenomena like taboo behaviors or rare events are difficult to capture. A thoughtful protocol can preempt Institutional Review Board concerns.

**Innovation:**

This novel guide provides a practical adaptation of traditional approaches to observation to meet contemporary healthcare research teams’ needs.

## Highlights

• Health research study designs benefit from observations of behaviors and contexts
• Direct observation methods have a long history in the social sciences
• Social science approaches should be adapted for health researchers’ unique needs
• Health research observations should be feasible, well-defined and piloted
• Multidisciplinary teams, data collection tools and detailed protocols enhance rigor

## Graphical abstract

**Figure:**
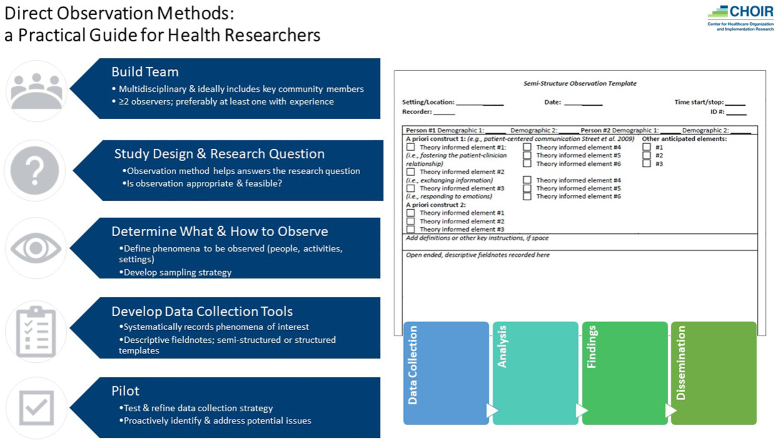

## 1 Introduction

Health research studies increasingly include direct observation methods [[1], [2], [3], [4], [5]]. Observation provides unique information about human behavior related to healthcare processes, events, norms and social context. Behavior is difficult to study; it is often unconscious or susceptible to self-report biases. Interviews or surveys are limited to what participants share. Observation is particularly useful for understanding patients’, providers’ or other key communities’ experiences because it provides an “emic,” insider perspective and lends itself to topics like patient-centered care research [1,5,6]. This insider perspective allows researchers to understand end users’ experiences of a problem. For example, patients may be viewed as “non-compliant,” while observations can reveal daily lived experiences that impede adherence to recommended care [[7], [8], [9], [10]]. Observation can examine the organization and structure of healthcare delivery in ways that are different from, and complementary to, methods like surveys, interviews, or database reviews. However, there is limited guidance for health researchers on how to use observation.

Observation has a long history in the social sciences, with participant observation as a defining feature of ethnography [[11], [12], [13]]. Observation in healthcare research differs from the social sciences. Traditional social science research may be conducted by a single individual, while healthcare research is often conducted by multidisciplinary teams. In social science studies, extended time in the field is expected [11]. In contrast, healthcare research timelines are often compressed and conducted contemporaneous with other work. Compared to social science research questions, healthcare studies are typically targeted with narrowly defined parameters.

These disciplinary differences may pose challenges for healthcare researchers interested in using observation. Given observation’s history in the social sciences there is a need to tailor observation to the healthcare context, with attention to the dynamics and needs of the research team. This paper provides contemporary healthcare research teams a practical, methodologically rigorous guide on when and how to conduct observation.

## 2 Methods

This article synthesizes the authors’ experiences conducting observation in social science and health services research studies, key literature and experiences teaching observation. The authors have diverse training in anthropology (GF, MM), systems engineering (BK) and psychology (MR). To develop this guide, we reflected on our own experiences, identified literature in our respective fields, found common considerations across these, and had consensus-reaching discussions. We compiled this information into a format initially delivered through courses, workshops, and conferences. In keeping with this pedagogical approach, the format below follows the linear process of study development.

## 3 Results

**Fig. 1:**
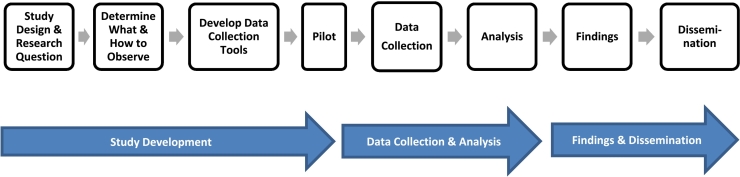
Direct observation across a health research study.

Following the trajectory of a typical health research project, from study development through data collection, analysis and dissemination (Fig. 1), we describe how to design and conduct observation in healthcare related settings. We conclude with data analysis, dissemination of findings, and other key guidance. Importantly, while illustrated as a linear process, many steps inform each other. For example, analysis and dissemination, can inform data collection.

### 3.1 Study development

#### 3.1.1 Study design and research questions

In developing research using observation, the first step is determining if observation is appropriate. Observation is ideal for studies about naturally occurring behaviors, actions, or events. These include explorations of patient or provider behaviors, interactions, teamwork, clinical processes, or spatial arrangements. The phenomena must be feasible to collect. Sensitive or taboo topics like substance use or sexual practices are better suited to other approaches, like one-on-one interviews or anonymous surveys. Additionally, the phenomena must occur frequently enough to be captured. Trying to observe rare events requires considerable time while yielding little data. Early in the study design process, the scope and resources should be considered. The project budget and the timeline need to account for staffing, designing data collection tools, and pilot testing.

Research questions establish the study goals and inform the methods to accomplish them. In a study examining patients’ experiences of recovery from open heart surgery, the ethnographic study design included medical record data, in-depth interviews, surveys, and observations of patients in their homes, collected over three months following surgery [7]. By observing patients in their homes GF saw how the household shaped post-surgical diet and exercise. Table 1 provides additional examples of healthcare studies using observation, often as part of a larger, mixed-method design [14,15].

**Table 1:** Example studies that use observation.

Research Topic	Study Design	Use of Observation
Organization, structure and process of HIV care.	Mixed Methods (survey, interviews and observation)	Site visits with observations of clinical encounters and staff work routines [1,16]
Identification of contextual factors influential in the uptake and spread of an anticoagulation improvement initiative.	Mixed Methods (survey, interviews, observation, and Interrupted time series)	Observations of clinical processes and clinical encounters with patients and of site champion quality improvement team meetings [4]
Examination of how physicians respond verbally and nonverbally to patient pain cues.	Observation of clinical interactions	Observations of clinical encounters [17,18]
Determination of proportion of tasks that are commonly carried out by clinical pharmacists can be appropriately managed by clinical pharmacy technicians.	Mixed Methods (modified Delphi process and observation)	Observation of pharmacists carrying out work tasks in a time-motion study [19]

#### 3.1.2 Data collection procedures

The phenomena to observe should be clearly defined. Research team discussions create a unified understanding of the phenomena, clarify what to observe and record, and ensure data collection consistency. This explication specifies what to look for during observation. For example, a team might operationalize the concept of *patient-centered care* into specific actions, like how the provider greets the patient. Further, additional nuances within broader domains (e.g., patient-centered care) could be identified while observations are ongoing. The team may identify unanticipated ways that providers enact patient-centered care (e.g., raising non-clinical, but relevant psychosocial topics- like vacations or hobbies- prior to gathering biomedical information). It is also important to look for negative instances, or behaviors that did not happen that should have, or surprising, unexpected findings. A surprise finding during observation was the impetus for further analysis examining how HIV providers think about their patients. While observing HIV care, a provider made an unexpected, judgmental comment about patients who seek pre-exposure prophylaxis (PrEP) to prevent HIV. This statement was documented in the fieldnotes (see 3.1.3 for a further description of fieldnotes) and later discussed with the team, leading to review of other study data and an eventual paper (see Fix et al 2018) [1]. Leaving room, both literally on the template and conceptually, can provide space for new, unexpected insights.

The sampling strategy outlines the frequency and duration of what is observed and recorded. It requires determining the unit of observation and the observation period. Units of observation are sometimes called “slices” of data. Ambady and Rosenthal [20] coined the term thin slices, using brief exposures of behavior (6s, 15s, and 30s) to predict teacher effectiveness. While thin slices are predominantly used in psychology, healthcare researchers can apply this concept by recording data for set blocks of time in a larger process, such as recording emergency department activity for the first 15 minutes of each hour.

The unit of observation can be a person (e.g., patient, provider), their behavior (e.g., smiling, eye rolling), an event (e.g., shift change) or interaction (e.g., clinical encounter). Using interactions as the unit of observation requires consideration for repeat observations of some individuals. For example, a fixed number of providers may be repeatedly observed with different patients.

Observation frequency will depend on the frequency of the phenomena. Enough data is needed for variation while also achieving “saturation,” a concept from qualitative methods, which means the point in data collection when no new information is obtained [21]. For quantitative studies, when examining the relationship between a direct observation measure (e.g., patient smiling) and an outcome (e.g., patient satisfaction), effect sizes from past research should dictate the number of interactions needed to achieve power to detect an effect. The duration of observation (the data slice) can be constrained using parameters as broad as a clinic workday, to distinct events like a clinical encounter.

Observation data can be collected on a continuous, rolling basis, or at predefined intervals. Continuous sampling is analogous to a motion picture—the recorded data mirrors the flow of information captured in a video [22]. Continuous observation is ideal for understanding what happens throughout an event. It is labor intensive and time-consuming and may result in a small number of observations, although each observation can yield considerable data. For example, a team may want to know about the patient-centeredness of patient-provider interactions. Continuous sampling of a clinical encounter could start when the patient arrives through when they leave, with detailed data collected about both the verbal and nonverbal communication. This could be considered an N of one observation but would yield substantial data. This information could be collected over a continuous day of encounters across several providers and patients, resulting in a considerable amount of data for a small group of people.

In contrast, instantaneous sampling can be conceptualized as snapshots, and is analogous to the thin slice methodology. Psychology research sometimes uses random intervals, while in healthcare research it may be preferable to use predetermined criteria or intervals [23]. Instantaneous sampling is economical and data collection can happen flexibly across a variety of individuals or times of day or weeks. Disadvantages include losing some of the context that is gained through continuous sampling.

#### 3.1.3 Data collection tools

Data collection tools enable systematic observations, codifying what to observe and record. These tools vary from open-ended to highly structured, depending on the research question(s) and what is known a priori. We describe below three general tool categories—descriptive fieldnotes, semi-structured templates, and structured templates.

##### 3.1.3.1 Descriptive fieldnotes

Descriptive fieldnotes, common in anthropology, are open-ended notes recorded with minimal a priori fields. Descriptive fieldnotes are ideal for research questions where less is known. An almost blank page is used to record the phenomena of interest. Key information such as date, time, location, people present and who recorded the information are useful for later analysis. These notes are jotted sequentially in real-time to maximize data collection, and are filled out and edited later for clarity and details. The flexible and open format facilitates the capture of unanticipated events or interactions.

Descriptive fieldnotes describe in detail what is observed (e.g., who is present, paraphrased statements), while leaving out interpretation. Analytic notes, that interpret what is being observed, can accompany the descriptive notes (e.g., the doctor is frowning and seems skeptical of what the patient is saying), but these analytic notes should be clearly marked as interpretation. One author (GF) demarcates interpretive portions of her fieldnotes using [closed brackets] to identify this portion of the fieldnote as distinct from the descriptive data. Interpretive notes should explain why the observer thinks this might be the case, using supporting data from the observation. Building on the example above, an accompanying interpretive note might say, “[the doctor raised their eyebrows, and does not seem to believe what the patient is saying, similar to what was observed in another encounter- see site 5 fieldnote). This information can be valuable during analysis to contextualize what was recorded and used in a later report or paper. Observation experience builds comfort and expertise with the open-ended, unstructured format.

##### 3.1.3.2 Semi-structured templates

**Fig 2:**
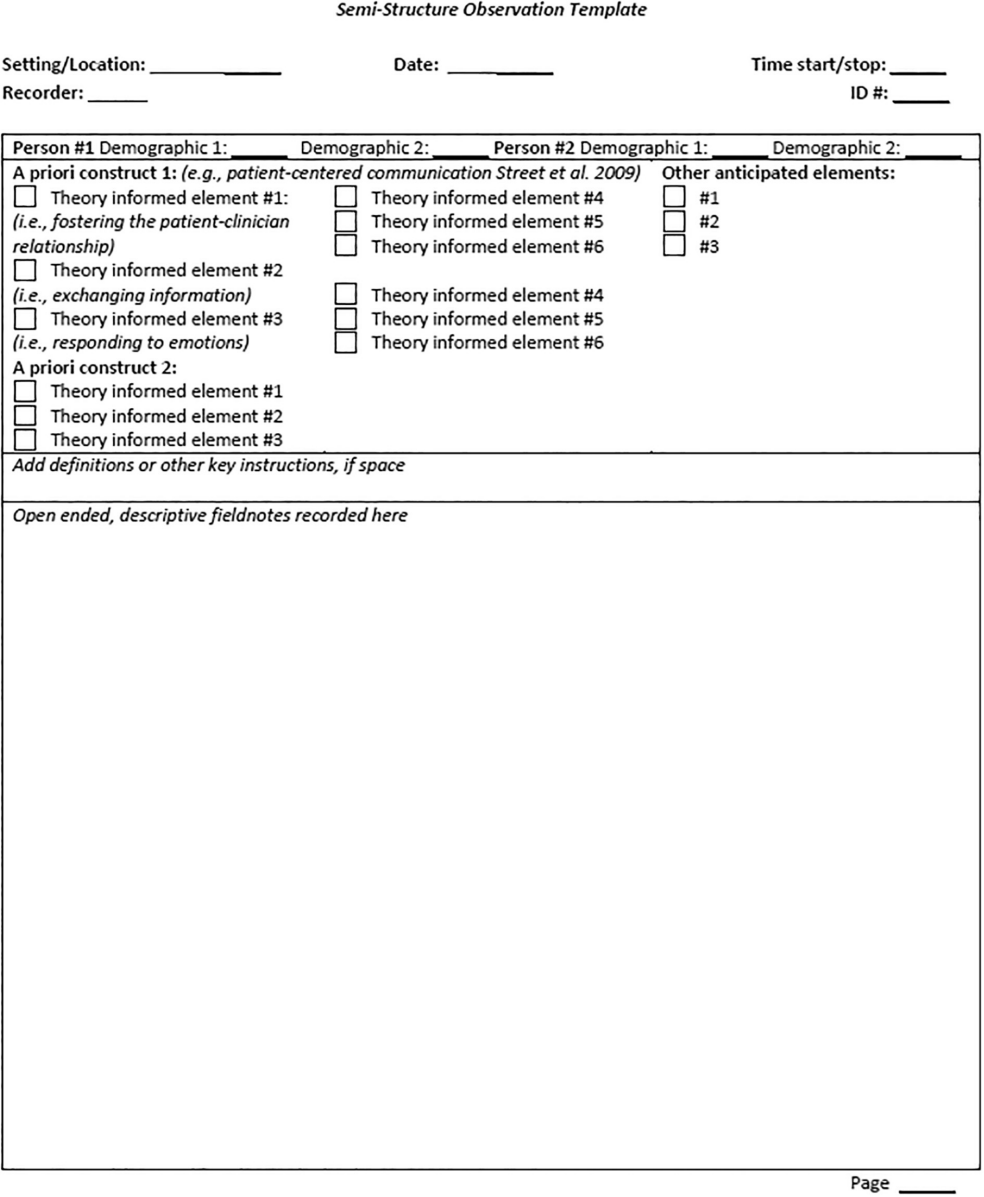
Semi-Structured Observation Template.

A semi-structured template comprises both open-ended and structured fields (Fig. 2). It includes the same key information described above (i.e., date, time, etc.), then provides prompts for a priori concepts underlying the research questions, often derived from a theoretical model. These literature-based, theoretical concepts should be clearly defined and operationalized. For example, drawing from Street et al’s [24] framework for patient-centered communication, we can use their six functions (fostering the patient-clinician relationship, exchanging information, responding to emotions, managing uncertainty, making decisions, and enabling self-management) to develop categories for semi-structured coding a template. Like descriptive fieldnotes, the template also provides open-ended space for capturing contextual details about the a priori data recorded in the structured section.

##### 3.1.3.3 Structured templates

A structured template in the form of a checklist or recording sheet captures specific, pre-determined phenomena. Structured templates are most useful when the phenomena are known. These templates are commonly used in psychology and engineering. Structured observations are more deductive and based on theoretical models or literature-based concepts. The template prompts the observer to record whether a phenomenon occurred, its frequency, and sometimes its duration or quality. See Keen [5] or Roter [25] for example structured templates for recording patient-centered care or patient-provider communication.

All templates should include key elements like the date, time and observer. Descriptive fieldnotes and semi-structured templates should be briefly filled out during the observation, and then written more thoroughly immediately afterwards. Setting aside time during data collection, such as a few hours at the end of each day, facilitates completion of this step. Recording information immediately, rather than weeks or months later, enhances data quality by minimizing recall bias. If written too much later, the recorder might fill in holes in their memory with inaccurate information. Further, small details, written while memories are fresh, may seem unremarkable but later provide critical insights.

For the semi-structured and structured templates, which contain prepopulated fields, there should be an accompanying “codebook” of definitions describing the parameters for each field. For example, building on the previous example using Street et al’s constructs, the code “responding to emotions” could identify instances where patients appear to be sad or worried and the provider responds to these emotions (also termed empathic opportunities and empathic responses) by eliciting, exploring, and validating the patients’ emotions [25,26]. This process operationally defines each concept and facilitates more reliable data capture. If space allows, the codebook can be included in the template and referenced during data collection. Codebooks should be updated through team discussion and as observations are piloted. Definitions from the codebook can be used in later reports and manuscripts.

### 3.2 Piloting

Given the real-world context within which observation data is collected, pilot-testing helps ensure that ideas work in practice. Piloting provides an opportunity to ensure the research plan works and reduce wasted resources. For example, piloting could reveal issues with the sampling plan (e.g., the phenomena do not happen frequently enough), staffing capacity (e.g., there are too many people to follow) or the codebook (e.g., few of the items specified in the data collection template are observed). Further, piloting gives the team a chance to systematize data collection and address issues before they interfere with the overall study integrity. This process guides what refinements need to be made to the data collection procedures. Piloting should be done at least once in a setting comparable to the intended setting.

### 3.3 Collecting data, analysis and dissemination

Healthcare studies are commonly conducted by interdisciplinary teams. The observation team should include at minimum two people, including someone with prior observation experience. Having more than one person collecting data increases capacity, distributes the workload and facilitates scheduling flexibility. Multiple observers complement each other’s perspectives and can provide diverse analytic insights. The observers should be engaged early in the research process. Having regular debriefing meetings during data collection ensures data quality and reliability in data collection. Adding key members of relevant communities to the team, such as patients or providers, can further enhance the relevance and help the research team think about the implications of the work.

Observational data collection often takes place in fast-paced clinical settings. For paper-based data collection, consolidating the materials on a clipboard and/or using colored papers or tabs, facilitates access. An electronic tablet to enter information directly bypasses the need for later, manual data entry.

Data analysis should be considered early in the research process. The analytic plan will be informed by both the principles of the epistemological tradition from which the overall study design is drawn and the research questions. Studies using observation are premised on a range of epistemological traditions. Analytical approaches, standards, and terminology differ between anthropologically informed qualitative observations recorded using descriptive fieldnotes versus structured, quantitative checklists premised on psychological or systems engineering principles. A full description of analysis is thus beyond the scope of this guide. Analytic strategies can be found in discipline-specific texts, such as Musante and DeWalt [27], anthropology; Suen and Ary [28], psychology; or Lopetegui et al [29], systems engineering. Regardless of discplinary tradition, analytic decisions should be made based on the study design, research question(s), and objective(s).

Dissemination is a key, final step of the research process. Observation data lends itself to a rich description of the phenomena of interest. In health research, this data is often part of a larger mixed methods study. The observation protocol should be described in a manuscript’s methods section; the results should report on what was observed. Similar to reporting of interview data, the observed data should include key descriptors germane to the research question, like actors, site number, or setting. See Fix et al [1] and McCullough et al [4] for examples on how to include semi-structured, qualitative observation data in a manuscript and Waisel et al [17] and Kuhn et al [19] for examples of reporting structured, quantitative data in a manuscript.

### 3.4 Institutional review boards

Healthcare Institutional Review Boards may be unfamiliar with observation. Being explicit about data collection can proactively address concerns. The protocol should detail which individuals will be observed, if and how they will be consented and what will and will not be recorded. Using a reference like the Health Insurance Portability and Accountability Act (HIPAA) identifiers (e.g., name, street address) can guide what identifiable information is collected. The protocol should also describe how the team will protect data, especially while in the field (e.g., “immediately after data collection, written informed consents will be taken to an office and locked in a filing cabinet”).

There are unique risks in studies using observation because data is collected in “the field.” Precautions attentive to these settings protect both participants and research team members. A detailed protocol should describe steps to address potential issues, including rare or distressing events, or what to do if a team member witnesses a clinical emergency or a participant discloses trauma. Additionally, team members may need to debrief after distressing experiences.

## 4 Discussion & conclusion

### 4.1 Discussion

The ability to improve healthcare is limited if real-world data are not taken into account. Observation methods can elucidate phenomena germane to healthcare’s most vexing problems. Considerable literature documents the discrepancy between what people report and their behavior [[30], [31], [32]]. Direct observation can provide important insights into human behavior. In their ethnographic evaluation of an HIV intervention, Evans and Lambert [31] found, “observation of actual intervention practices can reveal insights that may be hard for [participants] to articulate or difficult to pinpoint, and can highlight important points of divergence and convergence from intervention theory or planning documents.” Further, they saw ethnographic methods as a tool to understand “hidden” information in what they call “private contexts of practice.” While in Rich et al.’s work [32], asthmatic children were asked about exposure to smoking. Despite not reporting smoking in the home, videos recorded by the children—part of the study design—documented smokers *outside* their home. The use of observation can help explain research questions as diverse as patients’ health behaviors [7,10,32], healthcare delivery [3,4] or the outcomes of a clinical trial [9,33].

A common critique in healthcare research is that observing behavior will change behavior, a concept known as the Hawthorne Effect. Goodwin’s study [34], using direct observation of physician-patient interactions, explicitly examined this phenomena and found a limited effect. We authors have observed numerous instances of unexpected behavior of healthcare employees such as making disparaging comments about patients, eye rolling, or eating in sterile areas. Thus, those of us who conduct observation often say that if behavior change were as easy as observing people, we could simply place observers in problematic healthcare settings.

The descriptions above on how to use observation are applicable to fields like health services research and implementation and improvement sciences which have similarly adapted other social science approaches.[[35], [36], [37], [38], [39], [40]] Notably, unlike the social sciences, many health researchers work in teams and thus this guide is written for team-based work. Yet, health researchers sometimes also conduct observations without support from a larger team. While this may be done because of resource constraints, it may raise concerns about the validity of the observations. First, social sciences have a long history of solo researchers collecting and analyzing data, yielding robust, rigorous findings [13,[41], [42], [43]]. Using strategies, such as those outlined above (i.e., writing detailed, descriptive fieldnotes immediately; keeping interpretations separate from the data; looking for negative cases) can enhance rigor. Further, constructs like validity are rooted in quantitative, positivist epistemologies and need to be adapted for naturalistic study designs, like those that include direct observation [44].

### 4.2 Innovation

Social science-informed research designs, such as those that include observation, are needed to tackle the dynamic, complex, “wicked problem” that impede high quality healthcare [45]. Thoughtful, rigorous use of observation tailored to the unique context of healthcare can provide important insights into healthcare delivery problems and ultimately improve healthcare.

Additionally, observation provides several ways to involve key communities, like patients or providers, as participants. Observing patient participants can provide information about healthcare processes or structures, and inform research about patient experiences of care or the extent of patient-centeredness. With the movement towards engaging end users in research, these individuals can contribute more meaningfully [46,47]. As team members, they can define the problem, inform what to observe, how to observe, help interpret data and disseminate findings.

### 4.3 Conclusion

Observation’s long history in the social sciences provides a robust body of work with strategies that can be inform healthcare research. Yet, traditional social science approaches, such as extended, independent fieldwork may be untenable in healthcare settings. Thus, adapting social science approaches can better meet healthcare researchers’ needs.

This paper provides an innovative, yet practical adaptation of social science approaches to observation that can be feasibly used by health researchers. Team meetings, developing data collection tools and protocols, and piloting, each enhance study quality. During development, teams should determine if observation is an appropriate method. If so, the team should then discuss what and how to collect the data, as described above. Piloting improves data collection procedures. While many aspects of observation can be tailored to health research, analysis is informed by epistemological traditions. Having clear steps for health researchers to follow can increase the rigor or credibility of observation.

Rigorous utilization of observation can enrich healthcare research by adding unique insights into complex problems. This guide provides a practical adaptation of traditional approaches to observation to meet healthcare researchers’ needs and transform healthcare delivery.

## Funding

This work was supported by the US Department of Veterans Affairs, Veterans Health Administration, 10.13039/100006379Office of Research and Development, 10.13039/100007217Health Services Research and Development. Dr. Fix is a VA HSR&D Career Development awardee at the Bedford VA (CDA 14-156). Drs. Fix, Kim and McCullough are employed at the Center for Healthcare Organization and Implementation Research, where Dr. Ruben was a postdoctoral fellow. The authors received no financial support for the research, authorship, and/or publication of this article.

## Declaration of Competing Interest

All authors declared no conflict of interests.
